# Research priorities for rehabilitation and aging with HIV: a framework from the Canada-International HIV and Rehabilitation Research Collaborative (CIHRRC)

**DOI:** 10.1186/s12981-020-00280-5

**Published:** 2020-05-19

**Authors:** Kelly K. O’Brien, Francisco Ibáñez-Carrasco, Patricia Solomon, Richard Harding, Darren Brown, Puja Ahluwalia, Soo Chan Carusone, Larry Baxter, Charles Emlet, Gayle Restall, Alan Casey, Amrita Ahluwalia, Adria Quigley, Alex R. Terpstra, Nkem Ononiwu

**Affiliations:** 1 Department of Physical Therapy, University of Toronto, 500 University Avenue, Room 160, Toronto, ON Canada; 2 Institute of Health Policy, Management and Evaluation (IHPME), 155 College Street, 4th Floor, Toronto, ON Canada; 3 Rehabilitation Sciences Institute (RSI), University of Toronto, 500 University Avenue, Room 160, Toronto, ON Canada; 4 Centre for Urban Health Solutions, St. Michael’s Hospital, 30 Bond Street, Toronto, ON Canada; 5 School of Rehabilitation Science, McMaster University, 1400 Main Street West, Room 403, Hamilton, ON Canada; 6 Cicely Saunders Institute, King’s College London, Bessemer Road, London, UK; 7 Therapies Department, Chelsea and Westminster Hospital NHS Foundation Trust, London, UK; 8 Realize, 600 Bay Street, Suite 600, Toronto, ON Canada; 9 Casey House, 119 Isabella Street, Toronto, ON Canada; 10 Halifax, NS Canada; 11 University of Washington, Tacoma, Social Work, 1900 Commerce Street, Tacoma, WA USA; 12 College of Rehabilitation Sciences, University of Manitoba, R127 Rehab Building, Winnipeg, MB Canada; 13 Department of Physical Medicine and Rehabilitation, University of Manitoba, 820 Sherbrook Street, Winnipeg, MB Canada; 14 Fife House, 490 Sherbourne Street, Toronto, ON Canada; 15 Faculty of Health, Dalhousie University, 5968 College Street, Room 316, Halifax, NS Canada; 16 Department of Psychology, 2136 West Mall, Room 2405, Vancouver, BC Canada

**Keywords:** HIV, Rehabilitation, Aging, Disability, Research priorities

## Abstract

**Background:**

People living with HIV are living longer, and can experience physical, mental and social health challenges associated with aging and multimorbidity. Rehabilitation is well positioned to address disability and maximize healthy aging. An international collaborative network, called the Canada-International HIV and Rehabilitation Research Collaborative (CIHRRC), works to guide this emerging field. In this article, we report findings from CIHRRC’s aim to identify emerging research priorities in HIV, aging and rehabilitation from the perspectives of people living with HIV, clinicians, researchers, representatives from community organizations and policy stakeholders.

**Methods:**

We conducted a multi-stakeholder multi-method international consultation with people living with HIV, researchers, clinicians and representatives of community-based organizations to identify research priorities in HIV, aging and rehabilitation. Stakeholders identified research priorities during a one-day International Forum comprised of presentations and facilitated discussion. We collated and analyzed data using content analytical techniques, resulting in a framework of research priorities.

**Results:**

Sixty-nine stakeholders from countries including Canada (n = 62; 90%), the United Kingdom (n = 5; 7%), United States (n = 1; 1%) and Australia (n = 1; 1%) attended the International Forum on HIV, Aging and Rehabilitation Research. Stakeholders represented community-based organizations (n = 20; 29%), academic institutions (n = 18; 26%), community or institutional healthcare organizations (n = 11; 16%), research or knowledge production organizations (n = 10; 14%), and organizations representing government or industry (n = 10; 14%). The *Framework of Research Priorities in HIV, Aging and Rehabilitation* includes seven research priorities: (1) nature, extent and impact of disability, concurrent health conditions and chronic inflammation with HIV; (2) prevalence, severity and impact of frailty; (3) community and social participation aging with HIV; (4) strategies for chronic disease management and healthy aging with HIV; (5) facilitators and barriers to access and engagement in, rehabilitation; (6) effectiveness of rehabilitation interventions for healthy aging with HIV; and (7) advancing development and use of patient reported outcome measures in HIV and aging. The Framework highlights methodological considerations to approach the priorities and the importance of knowledge translation and exchange to apply research knowledge into practice, programs and policy.

**Conclusions:**

These priorities offer a foundation for collaboration among international and multidisciplinary teams to advance the field of HIV, aging and rehabilitation in order to promote healthy aging with HIV.

## Background

With universal access to effective and tolerable antiretroviral therapy, people living with HIV are living longer [1–3]. They are also presenting with new clinical and social challenges associated with aging [4]. In high income countries, the proportion of people living with HIV who are 50 years of age or older increased from 15% in 2000 to 33% in 2016, with a similar trend in low and middle income countries [5, 6]. As people living with HIV age, the rising prevalence of multimorbidity including cardiovascular disease, diabetes, bone and joint disorders, neurocognitive disorders, and more recently frailty, further add to the complexity of health challenges or disability, and increased health care needs, over the life course [7–16]. Adults aging with HIV can also face additional challenges of ageism, stigma, mental health challenges, income insecurity, and lack of social support, which may intersect and further compound issues of aging with HIV [17–21]. These health challenges may be conceptualized as ‘disability’, broadly defined as any physical, cognitive, mental and emotional and social health challenges that can be experienced as episodic in nature with periods of fluctuating health [22]. It is critical for researchers, clinicians and policy makers to understand the changing needs of people aging with HIV, to better address disability and to incorporate the role for rehabilitation [23, 24].

In an era where more people are living longer with HIV, it is important to approach health related well-being from the perspective of person-centered care, which is inclusive of rehabilitation. In 2015, the World Health Organization revised the definition of ‘healthy aging’ to “the process of redeveloping and maintaining functional ability that enables well-being in older age” recognizing the interaction between personal and environmental factors that influence health [25]. This definition moves away from the previously used term ‘successful aging’ and acknowledges the presence of chronic disease and resultant disability while focusing on maximizing one’s function and ability within their life experience [26]. However, interventions to maximize healthy aging and how to measure their impact remains less clear [25].

The Joint United Nations Programme on HIV/AIDS (UNAIDS) established a global target of “90-90-90” whereby 90% of all people living with HIV in a community or country will be aware of their HIV status, 90% of those aware will have initiated treatment, and 90% of those on treatment will achieve viral suppression [27]. Despite achievements in viral suppression, disability, such as fatigue, mental health challenges, and financial insecurity persist. People living with HIV report lower health-related quality of life compared to the general population [28]. Lazarus and colleagues (2016) proposed a fourth “90”, meaning that 90% of those with undetectable viral load should report good health-related quality of life [29]. This requires an integrated person-centered approach to care for people living with HIV that goes beyond viral suppression to consider multimorbidity and self-perceived quality of life [29]. In 2019, the UNAIDS subsequently adopted mental well-being as a fourth “90” illustrating the importance of taking into account the broader health domains aging with HIV. This demonstrated a shift towards the importance of considering mental health, and personal and environmental factors that influence well-being aging with HIV [30]. This shift was further emphasized by the recent Lancet HIV special series, launched on World AIDS Day 2019, focused on ‘HIV Outcomes Beyond Viral Suppression’ for living well with HIV [31].

Rehabilitation is well positioned to address the fourth “90”, as it involves the dynamic process involving prevention or treatment activities and services that address symptoms, functional limitations and social participation restrictions [23]. As people age with HIV and experience multimorbidity, the need for rehabilitation will increase as traditional rehabilitation services such as physical therapy (or physiotherapy) and occupational therapy can help to address physical, cognitive and mental health challenges, such as fatigue and difficulty with mobility and daily activities; enhance mental health; facilitate return to employment; and improve social participation [32–39]. Nevertheless, the field of HIV and aging is still emerging. Few people living with HIV access formal rehabilitation services [40]. While systematic review evidence demonstrates the cost-effectiveness of rehabilitation in preventing morbidity and mortality in chronic conditions, such as neurological, musculoskeletal, and cardiovascular disease [41–46], rehabilitation evidence specific to aging with HIV is still evolving [24].

Forming partnerships and exchanging knowledge with other countries where individuals experience similar challenges related to HIV and aging is essential to address research priorities in this emerging field. In 2009, we formed a *Canada*-*International HIV and Rehabilitation Research Collaborative (CIHRRC)*, a network of researchers, clinicians, people living with HIV, representatives from community organizations and policy stakeholders with an aim to translate knowledge and identify emerging priorities in HIV and rehabilitation research [47]. In 2013, members of this collaborative convened to develop research priorities in HIV, disability and rehabilitation that included: episodic health and disability; aging with HIV across the life span; concurrent health conditions; access to rehabilitation and models of rehabilitation service provision; effectiveness of rehabilitation interventions; and enhancing outcome measurement in HIV and rehabilitation research [48]. While these priorities provided a foundation from which to direct research and clinical efforts, they do not consider more recent and emerging rehabilitation issues specific to aging with HIV. As more individuals age with HIV and experience disability with complex multimorbidity, it is critical to consider a coordinated research response from the rehabilitation field to address disability, promote health and well-being and address social needs of people aging with HIV. Our aim was to identify research priorities in HIV, aging and rehabilitation from the perspectives of people living with HIV, clinicians, researchers, representatives from community organizations and policy stakeholders.

## Methods

We conducted a multi-stakeholder international consultation with people living with HIV, researchers, clinicians and representatives of community-based organizations. Stakeholders convened for a one-day *International Forum on HIV and Rehabilitation Research* in Winnipeg, Manitoba, Canada held in collaboration with the Canadian Association for HIV and Research (CAHR) [49] and *Realize,* a national HIV organization focused on advancing research, policy and practice for people living with HIV and other episodic conditions [50]. The objectives of the Forum were to: (1) facilitate knowledge transfer and exchange on HIV, aging and rehabilitation research, clinical practice, and service delivery; (2) establish new research and clinical partnerships; (3) foster mentorship and training in HIV, aging and rehabilitation research; and (4) identify emerging priorities in HIV, aging and rehabilitation [51]. Our focus in this report is on the research priorities that emerged from this consultation. We reviewed the need for ethics approval with the University of Toronto, HIV/AIDS Research Ethics Board who confirmed that given the consultative nature of the Forum, this work did not require ethics approval.

We invited people living with HIV, clinicians, academics, representatives from community-based organizations, community members, and all members of the Canada-International HIV and Rehabilitation Research Collaborative (CIHRRC) with interest and expertise in aging, HIV and rehabilitation. We promoted the Forum through email and website communications to members of *Realize*, CIHRRC, the Ontario HIV Treatment Network (OHTN) and Canadian Association for HIV Research (CAHR).

Nineteen invited speakers from Canada, the United Kingdom and United States presented on research and program evaluation related to HIV, aging and rehabilitation. The Forum included two Research Evidence Panel Sessions comprised of 11 presentations and one Plenary Panel Session with small and large group facilitated discussions integrated throughout. The first Research Evidence Panel Session was entitled: “Successful Aging with HIV and Multi-Morbidity” and the second Research Evidence Panel Session was entitled: “Rehabilitation Interventions and Strategies for Older Adults Living with HIV”. The Plenary Panel Session focused on strategies to bridge the gap between research and real world clinical and community practice and to identify research priorities in HIV, aging and rehabilitation. The Forum speakers’ presentation slides and videos are accessible here: http://cihrrc.hivandrehab.ca/2016-forum.php.

Data pertaining to stakeholder perspectives on research priorities were collected using the following five strategies:

- Prior to the Forum participants were asked to submit responses to the following questions, ‘*In your opinion, what are 2 new and emerging issues in the field of HIV, aging and rehabilitation?*’, and ‘*In your opinion, what are 2*–*3 key research priorities in the area of HIV, aging and rehabilitation that are essential for moving the field forward?*’ [strategy 1].
- During the Forum, participants were encouraged to document their ideas related to emerging research priorities using notepads [strategy 2], and three graduate student rapporteurs documented discussion during presentations and scheduled group discussion [strategy 3].
- At the end of the Forum, participants were asked to complete an evaluation form that included the following questions related to research priorities: ‘*What are the three most important “take*-*home messages” that you heard at the Forum?*’, and ‘*Are there topics or issues that were raised today that you would like to see covered in future Forums, workshops or webinars?’* [strategy 4]
- After the Forum, we met with student rapporteurs to consolidate key points related to research priorities on HIV, aging and rehabilitation that emerged from the Forum [strategy 5].

We used the collective responses, discussion, and feedback derived from these sources as the foundation for identifying the research priorities. We collated and analyzed the data using conventional content analytical techniques [52]. The primary author (KKO) reviewed all sources of data, coded and clustered codes into categories to represent research priorities in HIV, aging and rehabilitation. We used Microsoft Excel to organize the data and codes [53]. Members of a Core Team (KKO, FIC, PS, and a representative from *Realize*, Canada), met to review the data, identify research priority areas derived from the coding process, cluster the priority areas into broader content areas, and organize them into a draft Framework of Research Priorities. The Framework was circulated twice for review and refinement by members of the authorship team.

## Results

### Participant stakeholder characteristics

Sixty-nine stakeholders from Canada (90%; n = 62), the United Kingdom (7%; n = 5), United States (1%; n = 1) and Australia (1%; n = 1) with expertise in the field of HIV, aging with HIV and rehabilitation attended the Forum. Most were researchers (22%; n = 15), followed by educators (17%; n = 12), service providers (13%; n = 9), community members, people living with HIV and other chronic illnesses (6%; n = 4), graduate students (13%; n = 9), clinicians (6%; n = 4), and other stakeholders including coordinators and program managers (23%; n = 16). Researchers, clinicians and educators were primarily rehabilitation professionals (physiotherapists or occupational therapists), physicians (geriatrics, rehabilitation medicine, infectious diseases, psychiatry), and nursing. Stakeholders worked in community-based organizations (29%; n = 20), academic institutions (26%; n = 18), community or institutional healthcare organizations (16%; n = 11), research or knowledge production organizations (14%; n = 10), and organizations representing government or industry (14%; n = 10). Of the 69 stakeholders, 16 (23%) were speakers at the Forum; of these 13 (81%) were from Canada, two (13%) from the United Kingdom, and one (6%) from the United States.

### Framework of Research Priorities in HIV, Aging and Rehabilitation

**Fig. 1:**
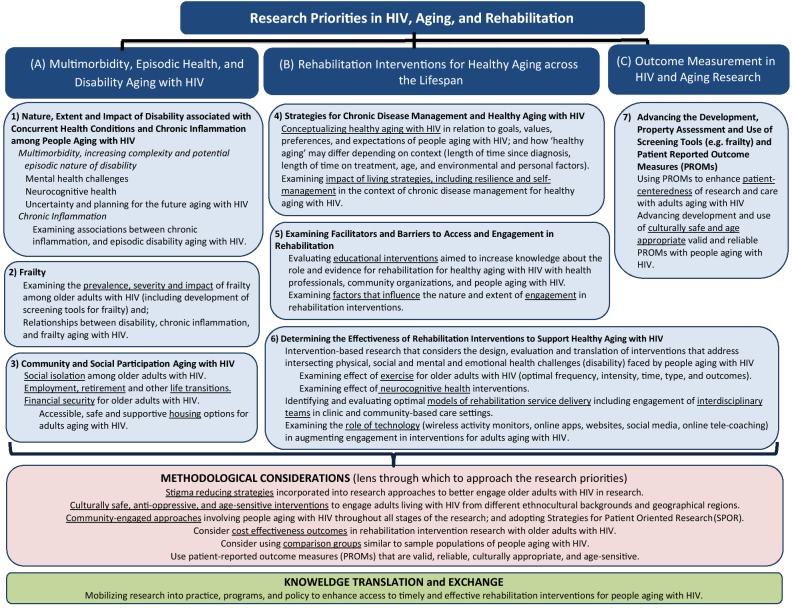
Framework of Research Priorities in HIV, Aging and Rehabilitation

The “*Framework of Research Priorities in HIV, Aging and Rehabilitation*” reflects how rehabilitation interventions have a critical role in addressing the complex health and social challenges experienced by individuals as they age with HIV and multimorbidity. It highlights priorities for HIV, aging and rehabilitation research, and offers a scaffold for collaboration among multidisciplinary teams to generate evidence on healthy aging with HIV. The Framework is comprised of seven research priorities: (1) examining the nature, extent and impact of disability resulting from concurrent health conditions and chronic inflammation aging with HIV; (2) examining the prevalence, severity and impact of frailty; (3) exploring community and social participation aging with HIV; (4) identifying strategies for chronic disease management and healthy aging with HIV; (5) examining facilitators and barriers to access and engagement in, rehabilitation; (6) determining the effectiveness of rehabilitation interventions to support healthy aging with HIV; and (7) advancing the development, measurement property assessment (validity, reliability, responsiveness), and use of screening tools and patient reported outcome measures (PROMs) in HIV and aging research (Fig. 1). These priorities were clustered into three broader content areas: (A) Multimorbidity, Episodic Health and Disability Aging with HIV; (B) Rehabilitation Interventions for Healthy Aging across the Lifespan; and (C) Outcome Measurement in HIV and Aging Research. The Framework includes methodological considerations identified from the consultation through which to approach the priorities, and highlights the importance of knowledge translation and exchange to mobilize research evidence into future practice, programs and policy (Fig. 1). The Framework is intended to inform future HIV, aging and rehabilitation research and to serve as a knowledge transfer and exchange tool that may be used by researchers, clinicians, students, people living with HIV and the broader HIV community. The priorities are presented below in no particular order of importance.

#### Content area 1: multimorbidity, episodic health and disability

Stakeholders highlighted the importance of multimorbidity prevention and health promotion as people age with HIV. This includes people living with HIV at all ages, and particularly older adults experiencing challenges due to the impact of years living with HIV, side effects of antiretroviral medications, and adults diagnosed with HIV in older adulthood.

### Research priority 1: nature, extent and impact of disability associated with concurrent health conditions and chronic inflammation among people aging with HIV

Research should seek to understand the prevalence, severity and impact of disability [22], including experiences of people aging with HIV and the added complexities that come from living with concurrent health conditions. Mental and cognitive health challenges such as depression, anxiety, and HIV-associated neurocognitive disorder (HAND) should also be considered. Concurrent health conditions experienced by people aging with HIV can be episodic in nature whereby the duration and intensity of illness and its resulting disability are unknown. Uncertainty was a specific domain of disability highlighted as a priority for people aging with HIV as a consequence of episodic illness, affecting stable employment and housing, and imposing restrictions on social engagement.

Stakeholders also highlighted the importance of exploring disability associated with inflammation and aging with HIV. Inflammation may increase risk for metabolic, bone, or cardiovascular health conditions [54–56]. Researchers should examine the association between chronic inflammation and disability among individuals aging with HIV; however, to our knowledge, there is a dearth of research considering the association between the inflammatory processes and daily physical and cognitive function with HIV. Better understanding the effects of chronic inflammation on disability aging with HIV can assist health care providers to develop targeted interventions aimed to manage disability over time.

### Research priority 2: frailty

Frailty is defined as an age-related syndrome to characterize a loss of reserves (energy, physical ability, cognition, health) that can yield to increased vulnerability and susceptibility to adverse clinical outcomes, such as hospitalization and disability [57, 58]. Stakeholders highlighted frailty as an emerging priority for adults aging with HIV. Research should examine the prevalence, severity and impact of frailty among adults aging with HIV, and the association between frailty and domains of disability (physical, mental, social and uncertainty domains) to develop strategies to prevent or minimize frailty among people living with HIV.

### Research priority 3: community and social participation aging with HIV

Stakeholders indicated how those diagnosed prior to the era of combination antiretroviral therapy might now be transitioning into retirement age with a history of unemployment and limited income support. This can result in unstable housing, and added stress and anxiety, subsequently limiting engagement with others in social settings and resulting in social isolation [59, 60]. In addition, stigma associated with HIV and aging emerged as an important contextual factor that may interact with gender, employment status, and ethnocultural background, further exacerbating challenges to community and social participation. Research should consider the experiences of people living with HIV as they transition into older age, and how differences in timing of HIV diagnosis in the pre or post combination antiretroviral therapy era can result in differences in community and social participation. Further research should seek to better understand the unique housing and social engagement needs of adults aging with HIV and multimorbidity. Researchers should collaborate with policy stakeholders and housing service providers to explore creative evidence-informed solutions to increase accessibility and affordable housing for adults aging with HIV.

#### Content area 2: rehabilitation interventions for healthy aging across the lifespan

Stakeholders emphasized the importance of rehabilitation interventions to address and prevent disability associated with aging with HIV and multimorbidity. Three research priorities were identified in this content area.

### Research priority 4: strategies for chronic disease management and healthy aging with HIV

Stakeholders highlighted the importance of identifying factors that facilitate healthy aging with HIV. This requires recognizing that goals, values, preferences and expectations may differ depending on age and length of time living with HIV. Self-management can be defined as “an individual’s ability to manage the symptoms, treatment, physical and psychosocial consequences living with a chronic condition” [61]. In the Forum discussion, self-management strategies emerged as a key component of healthy aging along with resilience, characterized as the ability to recover from longstanding episodic and chronic challenges living with HIV. Rehabilitation interventions that address the multidimensional and episodic nature of disability can reinforce and promote self-management skills for disability associated with HIV and aging [23, 62, 63]. Goal setting, promoting independence, enhancing recovery, recognizing progress, and valuing reassurance from others are key aspects of self-management support, and integral to developing self-efficacy through the rehabilitation process among people with chronic disease [64]. However, to date there is a paucity of research that examines models of health care that integrate these approaches specifically for older adults living with HIV. Future research should examine the impact of adopting living strategies, including resilience and self-management in the context of chronic disease management to promote healthy aging with HIV.

### Research priority 5: examining facilitators and barriers to access and engagement in rehabilitation for people aging with HIV

The role and importance of rehabilitation for healthy aging with HIV emerged from the consultation; however, stakeholders highlighted barriers that exist in accessing formalized services. Navigating the healthcare system for people living with HIV can be challenging with other competing life priorities (e.g., food, housing). Developing coordinated access to rehabilitation services through partnerships between healthcare providers and community-based organizations are integral for helping to navigate the system and identify where people aging with HIV may access services. For those with access to rehabilitation services, stakeholders highlighted the need to examine the nature and extent to which people living with HIV engage in rehabilitation and self-management interventions, such as physical activity and exercise.

Stakeholders further highlighted the need for evaluating the effect of educational interventions and strategies aimed to increase knowledge about the role of rehabilitation among current and future health professionals, recreation and community providers, and people aging with HIV. At *Realize*, connections are facilitated between HIV organizations and universities through role-emerging placements for physiotherapy and occupational therapy students [65]. Members of this team have developed educational modules for people living with HIV and rehabilitation professionals to enhance knowledge, skills and attitudes working in HIV care [66, 67]. Future research may examine the impact of new models of rehabilitation service delivery including interdisciplinary educational interventions on their ability to enhance knowledge and access to rehabilitation interventions for people living with HIV.

### Research priority 6: determining the effectiveness of rehabilitation interventions to support healthy aging with HIV

Stakeholders identified the need to evaluate the effect and translation of rehabilitation interventions to address the intersecting physical, social and mental health domains of disability experienced by adults aging with HIV. Rehabilitation interventions under evaluation should take into account the social determinants of health and diversity of populations of adults aging with HIV related to gender, social roles, age, duration of time living with HIV, literacy, multimorbidity, culture, race, geographic location and access to health services. It is important to understand the complexity of health issues faced by people living with HIV when evaluating rehabilitation interventions and models of rehabilitation service delivery [32, 33]. People aging with HIV should be actively involved in the planning of rehabilitation interventions to ensure approaches align with person-centered goals, values, preferences and diversity of the target audience. Physiotherapy is important for improving locomotor performance, strength, health related quality of life, and flexibility [37]. As the need for rehabilitation emerges and community health centers and clinics integrate physiotherapists and occupational therapists as members of the interdisciplinary team, there is an opportunity to maximize timely, appropriate and effective implementation and evaluation of rehabilitation services and interventions with the potential to optimize health outcomes for people living with HIV. Finally, as the role for rehabilitation continues to grow in the context of HIV and aging, stakeholders highlighted the importance of examining the need for evaluating the role of technology such as wireless physical activity monitors, online applications, social media, and online tele-coaching in measuring and augmenting engagement in rehabilitation.

#### Content area 3: outcome measurement in HIV and aging research

In addressing priorities related to episodic disability and the effect of rehabilitation interventions and education, stakeholders highlighted the need to advance outcome measurement in HIV and aging research, specifically developing and assessing tools for their ability to accurately and reliably measure indicators of health and disability for adults aging with HIV. We highlight a few specific constructs highlighted as important to HIV, rehabilitation and aging.

### Research priority 7: advancing the development, property assessment, and use of screening tools and Patient Reported Outcome Measures (PROMs)

Stakeholders identified the need to develop and advance HIV-specific person-centred screening tools and outcome measures to facilitate assessment and evaluate the effectiveness of interventions. Accurately and reliably screening for frailty was highlighted as a priority among adults aging with HIV to pre-emptively target strategies to prevent further progression. Measurements of frailty such as the Frailty Phenotype [57], Index [68], or Scale [69] exist; however with no gold standard assessment for HIV-associated frailty, there is a need to identify and validate which tools capture the presence and severity of frailty and can detect changes in frailty when it occurs among adults living with HIV. Stakeholders also emphasized the need to enhance patient-centeredness in research for older adults living with HIV by using Patient Reported Outcome Measures (PROMs) to evaluate disability experienced aging with HIV, facilitate communication between patients and providers, and examine the effectiveness of rehabilitation interventions.

### Methodological considerations

Our consultation process was not limited to research content areas alone. Methodological considerations for addressing these seven research priorities also emerged from the consultation. Stakeholders recommended that researchers consider barriers to engaging in research, such as stigma, and the need for culturally safe, anti-oppressive and age-sensitive interventions to better engage adults aging with HIV. For example, strategies for better engaging Indigenous adults aging with HIV in colonized countries such as Canada, new migrants living with HIV, individuals in rural geographical regions as well as those who may be experiencing stigma and fear of disclosure. Community-engaged approaches, involving people living with HIV in all aspects of the rehabilitation research is critical for ensuring the research is meaningful and relevant to the community [70–73]. Strategies such as the Strategy for Patient Oriented Research (SPOR) [74], and incorporating culturally appropriate, age-sensitive, valid, and reliable PROMs, were also recommended to yield better outcomes. Given the barriers to accessing rehabilitation in environments of fiscal restraint, researchers should include cost effectiveness outcomes in research evaluating rehabilitation interventions with older adults living with HIV. Other methodological considerations include considering the use of HIV-negative comparison groups, comprised of people matched in terms of age, gender and other important characteristics, to sample populations of people aging with HIV.

#### Knowledge translation and exchange

The final component of the Framework includes recommendations for translating research into practice, programs, and policy to enhance access to timely and effective rehabilitation interventions for people living with HIV. Stakeholders discussed the importance of linking research with practice, highlighting the necessity for research to be driven by the needs of communities of practice, and to ensure that research evidence is translated in a way to meaningfully impact programs and policy. Developing evidence-informed recommendations can facilitate translation of research into practice and optimize health outcomes for people aging with HIV. Members of our team established recommendations for rehabilitation among older adults with HIV, drawing on high-level evidence in other chronic conditions [24]. As new evidence emerges specific to rehabilitation interventions among people aging with HIV and multimorbidity, we will be able to revisit and enhance such recommendations incorporating HIV-specific literature. Strategies are also needed to facilitate the application of research knowledge generated from these priority areas. Mechanisms such as *International Forums on HIV and Rehabilitation Research* can facilitate translation of research evidence on HIV and rehabilitation in partnership with other annual conferences and providing open access to presentations and research findings [75] (http://cihrrc.hivandrehab.ca/forums.php).

## Discussion

The *Framework of Research Priorities in HIV, Aging and Rehabilitation* emerged from the perspectives of an international group of researchers, clinicians, people living with HIV, representatives from community-based organizations, funders and policy stakeholders in the field of HIV, aging, and rehabilitation. While the priorities are in no particular order, they outline a multi-directional path to examine disability and rehabilitation interventions, evaluating effectiveness with the use of PROMs. These priorities should not be addressed in isolation, but rather considered as overlapping constructs, such as the impact of episodic disability, and how rehabilitation interventions work in real world community or clinic-based settings. The Framework reflects research priorities specific to aging in the HIV and rehabilitation field, building on previous research priorities broadly established for HIV and rehabilitation [48]. As an international collaborative focused on HIV and rehabilitation research, we discuss how the field is striving to address these priority areas, and how this work provides a guide for further strengthening meaningful, rigorous and collaborative evidence on healthy aging in HIV and rehabilitation.

Members of our collaborative led foundational work examining reasons for referral, and effectiveness of rehabilitation interventions, specifically the role and impact of physiotherapy-led models of care for adults living with HIV in the United Kingdom [37], United States [62], Canada [32, 33] and South Africa [76–79]. Better understanding the biological, social and behavioural factors that interact with HIV and aging can help to identify effective rehabilitation interventions that promote well-being in this growing population [80]. The Episodic Disability Framework was developed from the perspectives of adults living with HIV, commonly used as a foundation for understanding how multimorbidity may interact with and influence health challenges over time aging with HIV [22, 81]. Solomon and colleagues used qualitative approaches to explore experiences of episodic disability over time, establishing phenotypes of episodic disability as a foundation in which to approach rehabilitation treatment for people aging with HIV and sometimes fluctuating multimorbidity [82]. While progress has been made towards understanding disability with HIV and aging, the majority of this work has been cross-sectional in nature. Future work to examine the types of disability domains that are experienced as episodic over time, and their magnitude of fluctuating severity will help to identify areas for providers to target interventions to mitigate or prevent episodes of disability among individuals aging with HIV.

Uncertainty and worrying about planning for the future is a key component of the *Episodic Disability Framework* [22] and emerged from our consultation as a priority for HIV, aging and rehabilitation research. Our earlier work demonstrated that uncertainty is a key feature of disability and strongly predicts mental health and social inclusion challenges as an individual ages with HIV [83, 84]. Older adults, representing the first cohort of people living with HIV to grow old, expressed concerns as to whether their health providers have age-related knowledge and skills to care for them as they age [85, 86]. Financial uncertainty is a concern, particularly for individuals diagnosed with HIV earlier in age who may have left the work force with no expectation of living into older adulthood, compared to those diagnosed later in life with years of pension contributions leading up to retirement [83]. For those working, employment not only provides financial benefits, but also a source of structure, social support, role-identity and meaning [87, 88]. As the fourth “90” of the UNAIDS “90-90-90” global targets concentrating on mental health and well-being, rehabilitation focused on strategies to engage people aging with HIV in community and social life, and evaluating the impact of these strategies, will be critical for people transitioning into retirement and longer term care settings. Examining the prevalence and impact of uncertainty, and determining how rehabilitation interventions can address worrying about the future and mental-emotional health challenges will be critical as adults age with HIV.

Loneliness and social isolation are key factors associated with depression, functional impairment and poorer quality of life among adults with HIV [89]. However, resilience (positive adaptation of past or present adversity) has been linked with healthy aging among adults living with HIV [90]; those who demonstrated high levels of self-acceptance and optimism, and implemented positive self-management strategies experienced health aging with HIV [91]. Positive self-management can result in better physical and emotional health, and health knowledge and behaviour among people living with HIV [92]. Members of the CIHRRC collaborative have made considerable strides examining the role of self-management interventions specific to people living with HIV [92]. Given the barriers to accessing formalized rehabilitation services, strategies promoting resilience and self-management have an increasing role for empowering individuals to maximize their own health and well-being while aging with HIV [85, 93]. For instance, exercise is a widely accepted rehabilitation intervention to improve physical and mental health outcomes among adults living with HIV [94–97] and implementation science approaches are underway to examine the effectiveness of community-based exercise for adults with HIV in Canada [98] and South Africa [77]. Evidence examining the effectiveness of yoga interventions also is emerging in the context of HIV [38, 99]; however, data comparing level of engagement in and response to these physical activity interventions among older versus younger adults living is limited. Ultimately, rehabilitation professionals should be included in multidisciplinary research teams in order to ensure safe and effective exercise prescription, and energy conservation and environmental modifications to prevent injury and sustain social participation to older adults with HIV, disability, frailty, and complex multimorbidity [58].

As more individuals age with HIV, geriatric syndromes such as frailty, fall risk, and declining physical function are increasingly important to measure and address within rehabilitation for adults aging with HIV [7, 100, 101]. The Fried phenotype [57] and Rockwood criteria [68, 102] have been commonly used in the HIV context [100] and new clinical guidelines outline ways to measure and treat frailty with people living with HIV [103]. Nevertheless, consideration of frailty in HIV and rehabilitation is still evolving. Researchers should more consistently consider frailty as an outcome of interest in rehabilitation research, better understand how to screen for pre-frailty, and tailor appropriate rehabilitation interventions to treat and prevent further progression.

Using PROMs in HIV care can foster a person-centred approach promoting patient involvement in decision-making, and improving communication and relationships between patients and professionals, which can encourage appropriate referrals, and improve treatment adherence [104]. Among 117 HIV-specific PROMs identified in a systematic review, most measured health-related quality of life (20%) with fewer capturing self-management (7%), stigma (7%), social support (3%) or disability (1%), all of which are important constructs to those aging with HIV [105]. Furthermore, approximately half of these measures were over 20 years old, and might not reflect the current health issues and contextual factors faced by adults aging with HIV. The development, validation and use of culturally sensitive, age- and contextually-appropriate measures that capture important constructs to adults aging with HIV diagnosed at different eras of antiretroviral therapy is needed to determine the effectiveness of rehabilitation interventions [104]. Members of our team developed the first known HIV-specific disability questionnaire, the HIV Disability Questionnaire (HDQ). This multidimensional tool has been validated for use among people living with HIV in Canada, Ireland, the United Kingdom and United States [106–108]. Current work is underway to develop and validate a short-form version of this PROM to enhance utility in the clinical setting. Communication among collaborative networks, such as CIHRRC may help to identify a common set of PROMs that may be used in research and practice in order to facilitate international cross-cultural comparisons of health outcomes and strengthen evidence in HIV, disability, and rehabilitation interventions.

These research priorities were developed from the perspectives of a multidisciplinary group of stakeholders with longstanding clinical, research and lived experiential expertise in HIV, aging and rehabilitation. Our community-engaged approaches involving people living with HIV in the consultation and development of the Framework was a strength given evidence suggests priorities can differ between health providers and patients living with HIV [109]. While we did not collect information on the age of stakeholders, older adults living with HIV were part of this consultation. We did not use a formal Delphi or nominal group technique to identify the priorities [110]. While our consultation was international in nature, stakeholders represented high income countries. The lack of representation of stakeholders from low to middle-income countries conducting work in HIV, aging and disability is a limitation of this work [15, 16, 76, 77]. Since this consultation, our international collaborative has grown to include partnerships with rehabilitation professionals in lower income countries such as South Africa, who have observed similar disability and rehabilitation issues in the context of HIV [79, 111]. While these priorities are specifically developed through a rehabilitation lens, addressing them will require collaborative and interprofessional and community-engaged approaches involving HIV, primary and geriatric care teams, social work, and psychology, in addition to rehabilitation to move the field forward. Members of our team are engaged in community-based and engaged methodological approaches and knowledge translation considerations in addressing these research priorities. For instance, Ibanez-Carrasco and colleagues challenged traditional methodology by working with peer researchers living with HIV and neurocognitive disorders in a Canadian study examining the impact of living with HIV associated neurocognitive disorders [112]. Finally, we acknowledge the field is continually changing and new priorities will emerge as the course of HIV evolves and the role for rehabilitation in the context of HIV continues to grow. Through mechanisms such as CIHRRC, we can facilitate new partnerships and translation of research evidence on HIV and rehabilitation providing open access to presentations and research findings and ongoing dialogue about new and emerging priorities in the field [75].

## Conclusion

As people live longer and age with HIV and the need for rehabilitation continues to rise, research addressing disability and effectiveness of rehabilitation interventions is critical for moving the field forward. We propose a *Framework of Research Priorities in HIV, Aging and Rehabilitation* comprised of seven priority areas in which researchers, clinicians, community members may build on foundational work to date to guide rigorous and meaningful evidence to inform the field. Examining strategies for chronic disease self-management, resilience effectiveness of rehabilitation intervention and advancing PROMs with adults living with HIV will be critical moving forward. These priorities outline a future plan for HIV, aging and rehabilitation research that will help increase our knowledge to enhance practice, programming and policy for people aging with HIV.

## Data Availability

The data used and/or analysed during the current study are available from the corresponding author on reasonable request.

## Abbreviations

CIHRRC: Canada-International HIV and Rehabilitation Research Collaborative
PROMs: Patient-reported outcome measures
UNAIDS: United Nations Programme on HIV/AIDS
